# Patient and public involvement in musculoskeletal pain research: using mutual learning to improve research quality

**DOI:** 10.1177/1759720X261436838

**Published:** 2026-04-24

**Authors:** Vasileios Georgopoulos, Stephanie L. Smith, Daniel F. McWilliams, Carolyn Desforges, Stevie Vanhegan, Kristina Staley

**Affiliations:** NIHR Nottingham Biomedical Research Centre, University of Nottingham, A25 Academic Rheumatology, Clinical Sciences Building, City Hospital, Nottingham NG5 1PB, UK; Academic Unit of Injury, Recovery and Inflammation Sciences, School of Medicine, University of Nottingham, Nottingham, UK; Arthritis UK Pain Centre, University of Nottingham, Nottingham, UK; NIHR Nottingham Biomedical Research Centre, University of Nottingham, Nottingham, UK; Academic Unit of Injury, Recovery and Inflammation Sciences, School of Medicine, University of Nottingham, Nottingham, UK; Arthritis UK Pain Centre, University of Nottingham, Nottingham, UK; NIHR Nottingham Biomedical Research Centre, University of Nottingham, Nottingham, UK; Academic Unit of Injury, Recovery and Inflammation Sciences, School of Medicine, University of Nottingham, Nottingham, UK; Arthritis UK Pain Centre, University of Nottingham, Nottingham, UK; Independent Consultant Arthritis UK Pain Centre, University of Nottingham, Nottingham, UK; Independent Consultant Arthritis UK Pain Centre, University of Nottingham, Nottingham, UK; TwoCan Associates, Ross on Wye, UK

**Keywords:** co-production, musculoskeletal pain, patient and public involvement, public partners

## Abstract

Public involvement is standard practice to enhance the quality, equity and impact of musculoskeletal pain research. In this review, we aimed to reflect on our own learning journey across multiple musculoskeletal projects and identify lessons learned from meaningful involvement. We then consider the changes needed at the researcher, funder and institutional levels to support involvement as a driver of relevance and impact. This is a narrative position paper drawing on experiential learning from three musculoskeletal pain studies and ongoing community engagement. We reflected on what we have learned from public contributors in our research, focusing on how involvement reshaped study priorities and tools. Lessons were synthesised to highlight recurring themes, supported by reference to reviews, guidance and empirical studies. Across all projects, several key themes emerged: (1) emphasis on purpose rather than process; (2) co-production and partnership rather than review; (3) flexibility and adaptation rather than predetermined steps; (4) relevant public contributors and partners; (5) focus on ‘learning’ rather than ‘doing’; and (6) approaches to diversity and inclusion. Public partnerships should be a collaborative, transformative, relational and learning-based process that reshapes all aspects of research. Realising this potential might require flexible funding for early engagement, training in facilitation and reflexivity, sustained support beyond the research project to promote impact, and taking steps beyond building more infrastructure towards strengthening the systems that enable involvement to work effectively.

## Plain language summary

Working together to improve muscle, joint, and bone pain research through shared learning

This paper looks at how public partners (patients, service users, carers and members of the public with relevant lived experience) are involved in research on conditions affecting pain, muscles, bones, and joints. Involving the public in research is now widely expected. Not only does this approach ensures that research genuinely reflects the priorities of the public, but it also shapes the research question and methods to ensure relevance, thereby increasing the likelihood that the findings are meaningful and usable in the real-world. It ensures that research is conducted for the benefit of the public with public input. Involvement is not always meaningful. From our experience, we found that when involvement was genuine and followed the principles of good practice, it not only reshaped the research and tool design but also provided clearer pathways to end-user benefit, underpinned by continuous two-way learning. As we reflect on working with public partners as equals in co-creating our research, it has not only shaped the research to ensure it aligns with our public partners’ priorities, but it has also shaped us as researchers. As researchers, we also face challenges: we must learn to negotiate with public partners as equals and need appropriate support, such as flexible funding, to respond effectively to their knowledge and expertise.

## Introduction

Patient and public involvement (PPI) is a term that collectively describes ways in which patients, service users, carers and members of the public with relevant lived experience work with researchers and healthcare professionals in the creation and use of healthcare research.^1^ PPI is an umbrella framework that ensures research is carried out with or by public contributors, rather than to, about, or for them. The perception of PPI has evolved from an optional ‘nice to have’ element to a recognised essential component of health research, and more recently towards genuine public partnerships – reflecting a progressive, relationship-based approach that emphasises shared decision-making and co-production. The benefits are particularly clear in musculoskeletal pain research, where conditions are often complex and multimorbid and where gaps in care remain substantial. For example, co-produced studies with people living with chronic pain^2,3^ and osteoarthritis^4,5^ have helped identify priorities that differ from those of clinicians – such as the need for more personalised support for self-management, improved communication around pain variability and better recognition of mental health impacts. Such examples illustrate how PPI can influence the design of rehabilitation programmes and inform service models that better reflect patient needs and lived realities.^6^ Evidence shows that when done well, PPI improves the quality, equity and impact of research.^7,8^ When meaningful, PPI can influence research priorities,^9,10^ study design^8^ and support the implementation of evidence-based guidance.^11^ These can be achieved by ensuring that research questions are relevant, study procedures are acceptable and findings are more likely to be translated into practice.^7,12,13^

Despite this evidence and the strong commitments of funders (e.g. National Institute for Health and Care Research (NIHR) and Arthritis UK), the quality of involvement remains variable.^14^ Many projects are still shaped by narrow understandings of involvement, resulting in tokenistic and inconsequential involvement.^15^ This variability is likely a combination of researcher inexperience, systemic barriers and different cultures, models and guidance.

Although public partnerships are designed to improve our research, they often also improve us as researchers. Through working with public contributors, we gain powerful insights that only people with lived experience can provide. This experiential learning reshapes our research, values, priorities and beliefs.^16^ It encourages us to think often outside the box, in how we communicate, ensure equal voices are heard and our contributors feel empowered.

In this article, we synthesise learning across three musculoskeletal pain projects (Thalidomide-Related Investigation on Understanding and Managing Pain for Thalidomide Survivors – TRIUMPH-TS, Central Aspects of Pain in Rheumatoid Arthritis – CAP-RA, Assessing Central Aspects of Pain – AsCent) and our ongoing work with community contributors. Our purpose is not simply to describe involvement activities, but to examine how sustained partnerships across projects reshape research priorities, methodological choices and interpretation within musculoskeletal pain research. By identifying recurring patterns across programmes, we make explicit the mechanisms through which public involvement strengthens epistemic validity, clinical relevance and implementation readiness in a field characterised by complex, multidimensional pain outcomes. In musculoskeletal pain research, where subjective experience, central sensitisation, functional disability and psychosocial context interact, decisions about measurement, interpretation and application carry implicit assumptions about what counts as valid knowledge. Sustained partnership provides a means of interrogating and refining those assumptions over time. We conclude by considering the structural and cultural conditions required at the researcher, funder and institutional levels to move involvement beyond compliance and towards embedded, practice-level change.

While the value of PPI is well established in health research,^7,8,16,17^ less attention has been given to how sustained partnerships across multiple projects reshape knowledge production within a specific clinical field. This paper synthesises longitudinal learning across three musculoskeletal pain projects and associated community engagement to identify recurring patterns in how ongoing partnership influences research priorities, methodological decisions and interpretation. In chronic musculoskeletal pain research, where subjective experience, central sensitisation, functional impact and psychosocial context intersect, decisions about measurement and application rest on assumptions about what constitutes valid and actionable knowledge. Our analysis makes visible how sustained public partnership challenges and refines these assumptions over time, strengthening epistemic robustness and implementation readiness. Our contribution, therefore, lies not in reiterating established PPI principles but in articulating how they operate within and respond to the specific epistemic and practical complexities of chronic musculoskeletal pain research and what structural conditions are required to embed this approach as routine practice.

## Approach to reflection and synthesis

This paper is a narrative review informed by structured reflection on PPI practice across three musculoskeletal pain research programmes (TRIUMPH-TS, CAP-RA and AsCent) and related community engagement activities (2021–2025). Reflections were based on documentary materials generated during these programmes, including PPI meeting notes, workshop outputs, annotated study documents revised following public feedback, internal debrief notes, email correspondence relating to design decisions and project reports. These materials were treated as practice-based records of how involvement influenced research processes and decisions.

Reflective discussions were undertaken by members of the research team and experienced public contributors involved in the programmes described. Reflection occurred iteratively at key project stages and was revisited during manuscript preparation. We identified specific examples where involvement led to changes in priorities, study design, language, interpretation or implementation planning.

Learning points were initially grouped into provisional categories and then compared across projects to map the current landscape of PPI in musculoskeletal pain and develop conceptual models of PPI in this field of research. Through iterative discussion and refinement, these categories were consolidated into six overarching themes representing shared learning across different contexts. Draft themes were circulated to contributing public partners for comment to ensure alignment with lived experience perspectives and to minimise researcher bias. Final themes were agreed upon through consensus.

This approach aligns with established principles of reflexive thematic synthesis and collaborative knowledge production in research with involvement.^7,8,16–18^ It was intended to enhance transparency and analytic rigour while retaining the reflective character of the paper.

## Reflections on our PPI experience in musculoskeletal pain research

When we first entered musculoskeletal pain research, our understanding of public involvement was primarily guided by published frameworks and funder templates. We recognised – and had personally witnessed – the value of involving people with lived experience. However, like many researchers with limited exposure to involvement practices, our early attempts at PPI did not reflect its intended multidimensional nature.^16^ Initially, our focus was procedural: establishing advisory groups, reviewing study materials and seeking feedback at key milestones. These activities fulfilled formal expectations but did not promote the deeper collaboration and shared ownership that meaningful involvement can bring. Over time, through various projects, we came to understand that PPI is not a checklist to complete but a relationship-based, mutually beneficial process that enriches both researchers and public contributors, leading to an enhanced research experience for all. The following examples outline key lessons learned across our projects, illustrating a steep learning curve that has profoundly reshaped how we now conduct research in genuine partnership with public contributors.

### Lessons from TRIUMPH-TS

Our learning curve became particularly evident during the TRIUMPH-TS project,^19^ which explored pain mechanisms in UK Thalidomide survivors (i.e., people born with limb deficiencies after their mothers were prescribed thalidomide during the first trimester of pregnancy). A defining feature of TRIUMPH-TS was the early and ongoing involvement of beneficiaries of the Thalidomide Trust. Before funding was secured, beneficiaries were invited to help shape the proposal and identify priorities for investigation. This early input led to the formation of a Beneficiary Advisory Group (BAG), which ensured that the study reflected issues that mattered most to the community – such as including carers’ and clinicians’ perspectives, and addressing outcomes related to medication use, mental health and fear of movement.

A particularly instructive example concerned the proposed use of the validated and publicly accessible Central Aspects of Pain (CAP) questionnaire^20–22^ to map pain distribution. The original version included a textbook body manikin, which beneficiaries felt could be distressing or inappropriate, as it was not reflective of the bodies of the people in their community. Their advice to replace the manikin with a tick-box list of body areas proved to be both acceptable to the wider community and effective in terms of participant engagement. This small but important change safeguarded participant comfort, improved data quality and demonstrated how early dialogue can identify and resolve potential barriers that might otherwise have affected recruitment or retention.

For the research team, this experience reaffirmed several key lessons. First, involvement is most valuable when it begins before the study design is fixed, allowing contributors to shape both content and approach. Second, experiential knowledge offers unique insights that can improve the rigour, acceptability and relevance of research methods – benefits well documented across health research.^31,32^ Third, meaningful PPI requires time, resources and mutual learning; it develops researchers’ ability to see issues from perspectives beyond their own disciplinary expertise.

In TRIUMPH-TS, sustained collaboration between researchers, clinicians and beneficiaries strengthened the scientific quality of the work and directly influenced the success of the project. The Thalidomide Trust commended the contribution approach, and reviewers highlighted it as a key strength during funding assessment. Beyond the immediate study, the process challenged us to view involvement as an integral component of research design rather than a discrete activity. More importantly, it provided a model for future projects within our group, showing how involvement can support both inclusion and methodological robustness. It underscored the value of mutual learning and reminded us that flexibility and openness are essential if involvement is to fulfil its potential to improve science as well as relevance.

### Lessons from central aspects of pain in rheumatoid arthritis study

In the Central Aspects of Pain in Rheumatoid Arthritis (CAP-RA) study,^20,23,24^ patients with rheumatoid arthritis (RA) played a pivotal role in reshaping the research focus and delivery. The study tested the validity of the CAP questionnaire in people with RA. The original self-report instrument^22^ was co-developed with public contributors with lived experience of persistent knee pain and was subsequently adapted for all joint pain. People with RA ensured the wording was appropriate to better reflect the condition’s variability and capture their lived experience. Although this shows the value of PPI in reviewing and approving study materials, it does not represent the full extent of the public’s influence on CAP-RA. The earliest approaches to public involvement for this study involved presentations made to groups of patients, where comments were invited. Although these were very useful in terms of face validity and participant burden, the largest single impact from PPI was from a less-structured discussion. Our initial plans were for a programme grant on RA pain, which was altered after listening to the testimonials of patients about the effects of fatigue on their lives. Highlighting what the researchers considered most important was different from patients’ experiences. Research plans evolved over time, and eventually the longitudinal, observational CAP-RA study was designed, which included a multidimensional measure of fatigue (Bristol Rheumatoid Arthritis Fatigue Scales – Multi-Dimensional Questionnaire: BRAF-MDQ), Short Message Service (SMS)-based reporting of the association between pain and fatigue, and pre-specified analyses that included fatigue. The important PPI discussions had been held across a routine working day, showing how providing scope for informal conversations around possible research can have a major impact, rather than relying only on formal meetings. Indeed, additional projects about fatigue in RA were initiated in response to this.^25^ Public contributors sat on the CAP-RA study steering committee and assisted in developing recruitment strategies and optimised the participant pathway through the two study visits, by rethinking data collection such as asking participants to complete questionnaires in the comfort of their own home, and reordering the visit to reduce it from 3 h to 90 min, thereby reducing participant burden, improving accessibility and uptake. A more collaborative approach was also used to determine study materials. A focus group of four people with RA analysed the meaningfulness of the CAP-RA study questionnaire, which was the primary data collection instrument. This group met in a hospital setting, familiar with the group members, and was jointly organised and co-led by a researcher and a person with RA.

CAP-RA shows how genuine collaboration can shift research from what researchers think is important to outcomes that resonate with patients’ daily lives. It highlights how being open to redirection ensures research is relevant and meaningful, rather than a tokenistic representation^11,13^.

### Lessons from AsCent study

The AsCent study was born from impromptu conversations with individuals from the CAP-RA study and public contributors highlighted a desire for improved pain management and diagnosis. Valuable insights from CAP-RA steering group members shaped both the design and delivery of the study, highlighting the importance of early and ongoing PPI. Public contributors felt that whilst the pain experience is unique to the individual, it is similar across multiple musculoskeletal conditions. This led to broader inclusion criteria, allowing for more people with musculoskeletal conditions to participate, making our sample more representative of real life. Valuable insights from CAP-RA also led to the decision for participants to complete the questionnaire booklet before attending study visits, reducing overall burden and improving engagement. Importantly, feedback provided during the later stages of a running project (CAP-RA) – when protocol changes were not feasible – was still used to inform future study planning, demonstrating that PPI contributions remain valuable beyond a project’s lifecycle. The project was co-produced with our public contributors from the project development and grant application stage through to their current roles on the steering committee. During co-production, as researchers, we are often acutely aware of the participant burden and trying to minimise this; however, it was our public contributors who were pushing for more granular data. This highlights that, by working together, we can find the balance between participants’ burden and obtaining rich, granular data to answer research questions that are relevant and meaningful to those who have most to gain from our findings.

Over time, working with our public contributors, we often take for granted the importance of their insights and the knowledge and experience they provide. Our contributors also want to feel like their input mattered and was meaningful. Through discussions with our public contributors and a formative process of co-production, we have developed a feedback system that allows them to see and understand how their contributions have shaped, refined and impacted the study. This involves documentation of key discussion topics and insights during meetings or informal interactions with our contributors, as well as the changes made as a result. This not only allows everyone to see the shared decision-making and valuable insights from our contributors, but it also enables us to catch any misinterpretations early.

Scientific publications are full of technical jargon, which our public contributors frequently tell us is difficult to understand, and they feel like they cannot contribute to. Based on this input, and for AsCent, we concurrently co-produce English-language summaries of our research, which we publish on our website. In response to our contributors’ suggestions and input, we are in the process of trying to publish these in journals alongside the peer-reviewed scientific article. This approach helped us realise that co-producing the English-language summary simultaneously with the scientific paper, rather than in retrospect, promoted a deeper understanding of the study and a clearer appreciation of its real-world impact.

### Lessons from our public contributors

For the Thalidomide Trust beneficiary involved in TRIUMPH-TS as a public contributor, the research delivered the appropriate outcomes because it was conducted with participants, not on or about them. Our contributor emphasised that studies designed solely because professionals or organisations believed they were ‘good’ for a community had often failed in the past, whereas TRIUMPH succeeded by co-producing the research from the outset. Beneficiaries were actively involved in shaping the proposal and setting the tone for authentic collaboration. It was further noted that meaningful co-production had required time, flexibility and shared passion from researchers and contributors alike. Although the process was empowering and affirming, it also demanded confidence to challenge assumptions and share ideas with academics and decision-makers. Engagement across the wider community had taken time and trust to build, as not all members were equally interested or motivated to participate. Nonetheless, being part of the research had reinforced a great sense of value and demonstrated how a respectful, inclusive partnership could produce outcomes that truly mattered to those affected.

Table 1 provides a concise summary of each programme, the forms of involvement undertaken and illustrative examples of how involvement influenced research decisions, mapped against the six overarching themes.

**Table 1.** Summary of programmes, involvement activities and illustrative impact mapped to overarching themes.

Programme	Nature of PPI involvement	Illustrative changes arising from involvement	Linked overarching themes
TRIUMPH-TS	Co-production workshops; BAG; iterative review of study documents; interpretation meetings	Refinement of research questions to reflect lived pain experience; modification of language in participant materials; adaptation of data collection procedures to reduce burden; co-development of dissemination outputs	Mutual learning; Shifting researcher assumptions; Designing with rather than for; Embedding lived experience in interpretation
CAP-RA	Ongoing PPI group input into study design and outcome measures; feedback on recruitment strategies; interpretation discussions	Adjustment of outcome measures to improve relevance; simplification of participant-facing documentation; refinement of recruitment messaging; contextualisation of findings in light of patient priorities	Relevance and rigour through involvement; Language and accessibility; Enhancing interpretive validity
AsCent	Early-stage involvement in conceptual development; co-production and management of project; stakeholder workshops; strategic discussions	Reframing of programme priorities; integration of implementation considerations at design stage; clarification of terminology and communication strategies	Involvement as strategic influence; Early partnership; Implementation-focused co-design
Public Contributors	Informal discussions; public events; feedback on dissemination materials	Identification of unmet needs; improved clarity in public-facing communication; shaping of future funding applications	Sustained relationships beyond projects; Learning as a reciprocal process

AsCent, assessing central nervous system contribution to accelerate musculoskeletal pain diagnosis and treatment; CAP-RA, Central Aspects of Pain in Rheumatoid Arthritis; PPI, patient and public involvement; TRIMPH-TS, thalidomide-related investigation on understanding and managing pain for thalidomide survivors.

## Discussion

Across our musculoskeletal projects, a consistent theme has been that involvement cannot be reduced to a set of predefined steps. Instead, it should be seen as a mutual learning model that evolves based on the context and contributor input rather than following rigid steps. This can only truly be achieved by embedding sustained co-production with context-specific networks where relationships have been built over time with individuals with relevant lived experience. Several themes have emerged around how involvement is conceived, resourced and sustained, and are discussed in detail below:

### From procedural compliance to purpose-driven involvement

There should be a move away from confirming that some PPI activity has occurred, to reporting how involvement has reshaped musculoskeletal pain research. Historically, much (but not all) musculoskeletal pain research has focused on a limited range of potential causative factors (often those which can be objectively measured). Lived experience has broadened our focus beyond symptom measurement to encompass participation, unpredictability, fatigue, work capacity, stigma and healthcare navigation. When PPI is reduced to procedural tasks (e.g. reviewing documents), it lacks the influence required to shape these deeper epistemic decisions. Our projects demonstrate that being open to meaningful involvement, through creating a safe space for learning, supports contributors in reshaping research and co-producing research that is meaningful to them. Training and funding guidance should encourage and support this purpose-driven model, emphasising how PPI may shift conceptual framing and opportunities for learning conversations throughout the entire research journey from inception to dissemination and beyond.

Guidance and reporting frameworks have been crucial in raising standards, such as the UK Standards for Public Involvement in Research,^26^ and when used correctly, should promote reflection and increase the impact of public contributors on the research process and outcomes,^8^ but should not be used as checklists. Involving public contributors and contributions is far more than checking that individuals agree with the researcher. Researchers are often uncertain about whom to involve and at what stage,^27^ with early engagement always encouraged; however, it can be difficult to know when to initiate and how. From our experience, impromptu conversations that lead to research questions and informal discussions with public contributors are a great place to start, and these relationships often develop into public partnerships.

Musculoskeletal pain-specific, purpose-driven involvement should shape the research question while hypotheses are being developed, enabling challenges to dominant research paradigms and influence outcome hierarchies that go beyond pain intensity. An interpretation informed by lived experience can expose and interrogate the epistemic choices underpinning musculoskeletal pain research, including what is measured, how outcomes are prioritised and how findings are framed, particularly in the context of the complexities of chronic musculoskeletal pain.

### From mere consultation and review to co-production and partnership

The focus should reposition public contributors in musculoskeletal pain research as co-producers in all aspects of the study, rather than validators of research-led design. Too often, involvement can be seen as a communication function – simplifying language in study documents or producing/approving lay summaries. In chronic musculoskeletal pain, the field grapples with epistemological uncertainty, stigma and contested aetiology. In such contexts, consultation is insufficient. Public contributors’ insights are invaluable, for both shaping and implementing studies, and we encourage researchers to utilise them further. Researchers often retain decision-making authority even within studies with extensive and innovative involvement. In musculoskeletal pain, where lived experience frequently challenges clinical orthodoxy, power asymmetry can blunt the transformative potential. Training and support around public contributors should really focus on co-production, embedded facilitation, reflexivity and power-sharing to ensure our public contributors’ voices are treated as equal. These experiences show that involvement should be understood as a mechanism to co-produce, not just review.

### From standard structures to context-sensitive models of involvement

Pain is a complex, multi-dimensional issue. Osteoarthritis in older adults, inflammatory arthritis, chronic widespread pain, post-surgical pain and pain in underserved communities carry different sociocultural meanings and healthcare trajectories. It is therefore important to replace default advisory group models with context-responsive approaches tailored to specific musculoskeletal populations.

Current funding and institutional models often encourage standardised approaches, such as establishing advisory groups, without sufficient regard for their composition, which can result in over-reliance on experienced contributors disconnected from specific lived realities. Advisory groups are useful, but careful consideration is required to select individuals with relevant lived experience or cultural relevance for the specific project/task. In TRIUMPH-TS, engagement required a bespoke BAG co-developed with survivors, which fed into the steering group. Culturally grounded partnerships require sustained investment in trust-building and flexibility across the research lifecycle. In musculoskeletal pain research, this is especially critical, as pain is inherently fluctuating and lived experience is dynamic rather than fixed; therefore, involvement must adapt alongside lived experience. These examples underline that flexibility and sensitivity to context are more important than replicating standard approaches.

Grant applications often require researchers to develop PPI plans at the outset. These plans should primarily be designed to enhance the project’s quality and impact. This can be facilitated by flexible, adaptable plans developed in consultation with our public contributors. Trying to stick to fixed plans can discourage learning and co-development, particularly in experiential learning cycles. Funding mechanisms in musculoskeletal research should allow for adaptive PPI plans with longer timelines to build trust, develop sustained relationships, but enable the flexibility to adapt with lived experiences and the evolving epistemology.

### From ‘participant-ready’ contributors to context-relevant partnerships

Carefully selecting people with relevant lived experience often requires building rapport, which takes time that should not be underestimated. Our experiences often suggest that establishing new networks can be a valuable adjunct to long-standing groups. Standing public contributor groups provide a pool of individuals who, over time, can develop into professional public contributors. These groups offer an opportunity to sustain relationships outside of individual projects and often have the necessary training already in place. However, over-reliance on ‘participant-ready’ individuals needs to be considered carefully. Experienced contributors can become strong patient leaders, offering valuable insights and helping to identify barriers within research processes and project delivery. Nonetheless, out of convenience or ease of access, standing groups can sometimes be asked to advise on topics beyond their lived or professional experience, which may reduce the relevance and impact of their input.^28^

Our experience suggests that authentic engagement often means building new networks tailored to context.^29^ This is more resource-intensive, as sustained involvement and continuous, contextually diverse presence are necessary, but ultimately will introduce new voices, diverse opinions and unique insights into the conversation. Institutions could help with this by acting as brokers to facilitate the connections between researchers and communities. Musculoskeletal pain is deeply shaped by work, gender roles, ageing and cultural meaning. Without contextually relevant voices, research risks reproducing narrow interpretations of burden and recovery. With fluctuating pain and fatigue associated with musculoskeletal pain, flexibility is required to support these partnerships. Institutions and musculoskeletal-oriented organisations can facilitate context-relevant partnerships by acting as brokers with community networks.

### From procedural involvement to learning-driven practice

Our projects showed that the most valuable outcomes of involvement were not quantifiable outputs but insights that reshaped the work. Reflecting and reporting on these reflections allows others to also learn from the process and for wider knowledge exchange. PPI in musculoskeletal pain research should demonstrate conceptual shifts, which may include reframing of priorities, changes in interpretation and altered dissemination narrative. These changes are rarely captured in reporting frameworks.

There are limited tools that assess the impact of PPI or reflect on learning. Reporting frameworks should encourage researchers to critically reflect on the journey and impact public contributors have had in terms of observable changes, especially to the research process.^30^ There needs to be a deliberate effort to find ways to embed reflection on the subjective learning experienced by researchers, for example, changes in values, assumptions, priorities and beliefs that are not yet an integral part of standardised reporting structures. As PPI gains recognition across academia, descriptions of its benefits should become more prominent in the discussion sections of published scientific reports to encourage learning and improve practices. As authorship is replaced by contribution statements, this provides another route for public contribution to be acknowledged in print. Reflexive documentation of the epistemic and conceptual impact of PPI, including how researchers’ assumptions have changed, how public contributors’ lived experience altered the methodological choices, reframed questions and reshaped interpretation, is far more valuable than descriptions of the process.

### From generic diversity principles to context-sensitive inclusion strategies

Every effort should be made to ensure that a diverse range of voices is heard, as this is crucial for making our research highly relevant not only to participant-ready individuals but to the population we aim to serve. We also need to be conscious of maintaining relevant lived experience. Too often, involvement privileges ‘participation-ready’ individuals while marginalising others.^12^ There is a necessity to include underserved groups, and sometimes trust can only be built through presence in community spaces, with contributors emphasising reciprocity rather than extraction. Diversity and inclusion should not be reduced to a general principle that includes individuals solely based on demographic characteristics, without relevant lived experience. This risks tokenism and undermines the authenticity of engagement. Instead, context-specific strategies, with adequate resourcing and ethical guidance on boundaries, are required.

Musculoskeletal pain disproportionately affects manual workers, older adults, women and people in socioeconomically deprived communities.^31^ Yet engagement with many of these groups is lacking, often drawing on those with time, resources and prior engagement experience. Authentic musculoskeletal PPI requires investing in trust-building within community settings, reciprocity rather than extractive engagement, recognition of fluctuating capacity due to pain and fatigue, and ethical clarity around boundaries and compensation. Musculoskeletal funding sources should explicitly resource community engagement infrastructure rather than expecting diversity without structural support.

### Moving beyond infrastructure

Over the past decade, substantial investment has gone into building PPI infrastructure across musculoskeletal research – through networks, advisory panels and toolkits (e.g. UK Standards for Public Involvement) that provide valuable foundations for involvement and our studies are prime examples of their application and utilisation. These structures have created opportunities for dialogue between researchers and public contributors, strengthened governance and enhanced awareness of involvement as a core component of research practice.^29^ However, infrastructure alone cannot guarantee meaningful participation or sustained relationships.

Funding often supports public involvement only for the duration of a specific project, leaving limited scope for the early relationship-building and post-project continuity that make involvement impactful. As discussed above, genuine partnerships require time, trust and flexibility – elements that rarely fit within short-term funding cycles. Researchers must therefore plan strategically to identify resources that enable engagement from the earliest stages through to dissemination and implementation.

The challenge now is to move more towards infrastructures that promote and strengthen the systems that enable effective learning-based PPI beyond the life course of the project. Repeated cycles of funding for new networks and panels risk duplicating effort and reinforcing procedural, compliance-driven approaches.^8,10^ Instead, targeted investment should prioritise mechanisms that sustain relationships across projects, provide flexible funding for early engagement and support researcher training and brokerage to connect with diverse communities.

Infrastructure remains an important enabler, but without parallel system-level change – including supportive funding, training and institutional recognition – involvement risks remaining a tick-box activity and a process to be completed rather than a shared endeavour that transforms research.

Taken together, we should be repositioning PPI in musculoskeletal pain research as a mechanism for epistemic transformation, in a field historically shaped by biomedical reductionism. Rather than asking whether PPI occurred, we should be asking whether involvement reshaped the question and what was measured, whether it challenged dominant assumptions, whether it redistributed decision-making power and whether it introduced perspectives that would otherwise remain absent. That is the move from involvement as a process to involvement as knowledge transformation.

### Limitations

This reflective synthesis is grounded in the experience of one musculoskeletal pain research group operating within a UK academic and NHS context. The institutional environment, funding structures and established relationships described may not be directly transferable to all settings. Public contributors involved in these programmes were relatively experienced partners, which may differ from contexts where involvement is less embedded or resourced.

The analysis is based on documentary materials and collaborative reflection rather than prospectively collected qualitative data. Although themes were refined with input from public partners, the synthesis remains shaped by the perspectives of those involved. The applicability of these themes to other clinical areas, health systems or resource contexts should therefore be interpreted with consideration of local circumstances.

### Pulling the threads together

Involvement should be understood as a mutually beneficial learning process rather than a fixed set of activities. Meaningful engagement moves towards co-development, supported by flexible approaches, researcher training, and policies that enable relationship-building, inclusion and continuity. These elements are central to enhancing research relevance, equity and impact.

To support this shift, universities, funders and research institutions must enable relational and learning activities through flexible funding, sustained engagement and recognition of experiential knowledge alongside scientific expertise.^32^ Framing involvement as co-development and mutual learning^16^ moves beyond compliance and towards embedded partnership. When aligned across the research system, this approach can strengthen innovation, relevance and impact within health research.

## Conclusion

Sustained PPI can move beyond consultation to reshape research practice when it is embedded across programmes rather than confined to individual studies. Our experience in musculoskeletal pain research shows that mutual learning, early partnership and iterative reflection translate established PPI principles into concrete changes in research design, interpretation and implementation. While grounded in a specific institutional context, this work demonstrates how long-term relationships can strengthen research relevance, rigour and clinical applicability. Embedding involvement as an ongoing partnership, rather than a discrete activity, is central to achieving practice-level change.

## Declarations

**Ethical approval and consent to participate:** No ethical approval was required for the purposes of this work. All aforementioned projects have received ethical approval from the appropriate authorities. Consent to participate was not applicable in this review.

**Consent for publication:** Not applicable.

**Availability of data and materials:** The datasets generated and/or analysed during the aforementioned studies are not publicly available due to copyright reasons but are available from Dr Vasileios Georgopoulos (Vasileios.georgopoulos@nottingham.ac.uk) and Dr Stephanie Smith (stephanie.smith2@nottingham.ac.uk) on reasonable request.
